# Prevalence of silicone oil droplets in eyes treated with intravitreal injection

**DOI:** 10.1186/s40942-019-0184-9

**Published:** 2019-09-11

**Authors:** Gustavo Barreto Melo, Celso de Souza Dias Junior, Fábio Barreto Morais, Alexandre Lima Cardoso, Ana Galrão Almeida Figueiredo, Acácio Alves Souza Lima Filho, Eduardo Büchele Rodrigues, Geoffrey Guy Emerson, Maurício Maia

**Affiliations:** 1 Hospital de Olhos de Sergipe, Rua Campo do Brito, 995 São José, Aracaju, SE 49020-380 Brazil; 2 Federal University of São Paulo, São Paulo, SP Brazil; 3 Tiradentes University-UNIT, Aracaju, SE Brazil; 4 Retina Center of Minnesota, Minneapolis, MN USA

**Keywords:** Ultrasonography, Intravitreal injection, Silicone oil droplets, Syringe

## Abstract

**Objective:**

To assess the number of eyes with silicone oil in the vitreous after intravitreal injection.

**Methods:**

This cross-sectional, comparative study was divided into 2 groups: (1) treatment—eyes subjected to antiangiogenic therapy; (2) control—no history of intravitreal injection. Subjects were assessed regarding age, gender, clinical diagnosis, lens status, visual acuity and number of previous intravitreal injections. All eyes underwent a meticulous slit-lamp and ultrasound examination for the identification of silicone oil. ImageJ software was used to quantify the index of silicone oil (IOS) by ultrasonography.

**Results:**

Sixty-seven eyes (30 controls, 37 treated) were included. Slit-lamp examination found silicone oil droplets in 25 out of 37 (67.57%) treated eyes and in none of the control group. Ultrasonography identified silicone oil in 28 out of 37 (75.68%) treated eyes and in 1 out of 30 (3.33%) controls. An observed agreement of 85.07% and a Cohen’s Kappa coefficient of 69.10% (p < 0.0001) between ultrasonography and biomicroscopy were found. Wilcoxon test showed a statistically significant difference (p = 0.0006) in IOS between controls (0.41 ± 0.43%) and treated eyes (2.69 ± 2.55%). Spearman’s correlation test (0.61; p < 0.0001) showed that the greater the number of injections, the higher the IOS.

**Conclusions:**

Silicone oil droplets were found in the majority of the eyes previously treated with antiangiogenic intravitreal injection. The greater the number of injections, the higher the likelihood of finding silicone oil. An improvement in the technique of injection and better-quality syringes post-injection silicone oil droplets.

## Background

Intravitreal injections are the most commonly performed intraocular treatment worldwide [1]. Until roughly a decade ago, they were administered to treat infectious endophthalmitis, inflammatory conditions and macular edema, as well as to inject gas tamponades for pneumatic retinopexy [2]. Since anti-vascular endothelial growth factor (VEGF) agents were found to be effective to treat age-related macular degeneration (AMD), the number of intravitreal injections has increased [3]. Nowadays, they are routinely used to treat AMD, macular edema secondary to diabetes or retinal vein occlusion, myopic choroidal neovascularization, and proliferative diabetic retinopathy [4, 5].

Recent studies have reported that silicone oil droplets might be released by the syringe [6–9]. Since many individuals complain of floaters, vitrectomy has been increasingly performed, despite risk of complications, such as retinal tears and detachment, vitreous hemorrhage, and endophthalmitis [10]. Such vision-threatening diseases should not be acceptable as secondary to the presence of silicone oil droplets in the vitreous.

Some studies also have reported that some medications are more prone to cause ocular inflammation than others [11–13]. However, the causes are uncertain. Some reports have suggested the possible role of syringes used during intravitreal injections [11, 14]. Our group carried out a case–control study that associated inflammation after intravitreal injection of aflibercept (Eylea, Regeneron Pharmaceuticals, Tarrytown, NY) with the use of a specific brand of syringe (Saldanha Rodrigues [SR], Manaus, Brazil) [14]. It was speculated that there was a possible link between aflibercept and the inflammatory response to the silicone oil droplets.

Therefore, the goal of the current study is to determine the prevalence of eyes with silicone oil in the vitreous after intravitreal injection and its association with the number of previous procedures.

## Methods

This was a cross-sectional, controlled study that was approved by the Institutional Review Board of the Federal University of Sergipe (CAAE 97505118.0.0000.5546). The tenets of the Declaration of Helsinki were followed and an informed consent was obtained from the subjects.

The individuals were divided into 2 groups:

- Treatment group: eyes previously subjected to an intravitreal injection of antiangiogenic by a single retina specialist (GBM) that consecutively presented for a routine evaluation;
- Control group: eyes without a history of intravitreal injection, either the contralateral untreated eye of one in the treatment group, or from a healthy subject (which could have both eyes included).

Subjects were assessed regarding age, gender, clinical diagnosis, lens status, best corrected visual acuity (BCVA) and number of previous intravitreal injections. All eyes underwent a meticulous slit-lamp and ultrasound examination for the identification of silicone oil droplets in the vitreous by experienced graders at the same office visit and a minimum of 7 days after the last injection.

Eyes with a prior history of vitreous hemorrhage, synchysis scintillans, asteroid hyalosis, or any vitreoretinal surgery were excluded because they could mistake the interpretation of the findings.

**Fig. 1:**
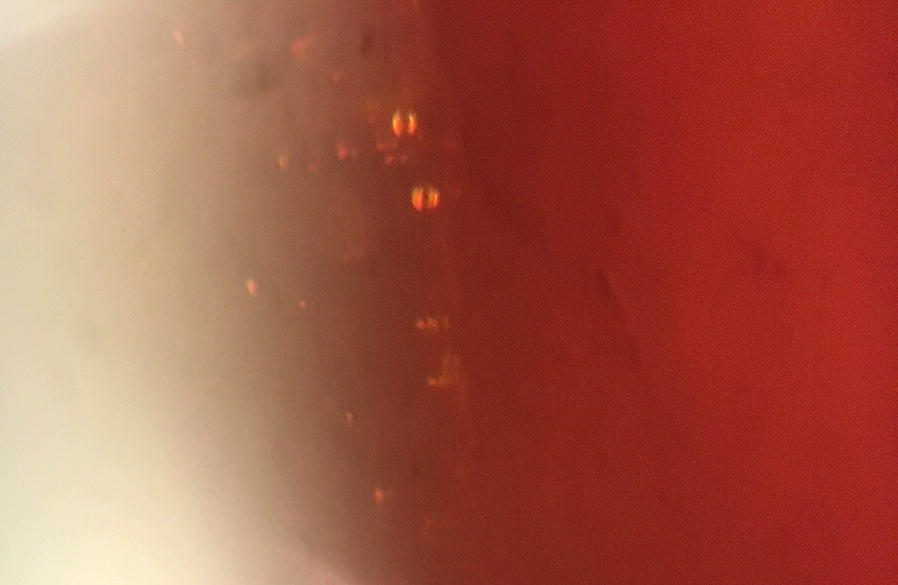
Silicone oil droplets in the anterior vitreous of one patient. Image captured on the slit-lamp with a smartphone mounted onto its ocular

Silicone oil droplets were identified in the slit-lamp examination by a single unmasked examiner (GBM) as a round, refractile and mobile substance (Fig. 1) either in the anterior vitreous by direct visualization or in the mid to posterior vitreous with the use of 78-D funduscopic lens. The same examination technique was applied to all treated and control eyes. Any amount of silicone oil was considered as positive.

B-scan ultrasonography was performed with a standard ultrasonograph and a 10-MHz transducer (EZ Scan AB5500+, Sonomed, NY, USA), with the patient lying supine. Since this technique is dynamic and operator dependent, the examiner was instructed to positively classify the presence of silicone oil based on the characteristic hyperreflective and mobile dotted appearance. Since other confounding factors, such as any vitreous disease as previously mentioned, had been excluded, the odds of misinterpretation were reduced. The B-scan image of the plane that disclosed the largest amount of hyperechoic droplets was recorded by the same masked examiner (FBM).

**Fig. 2:**
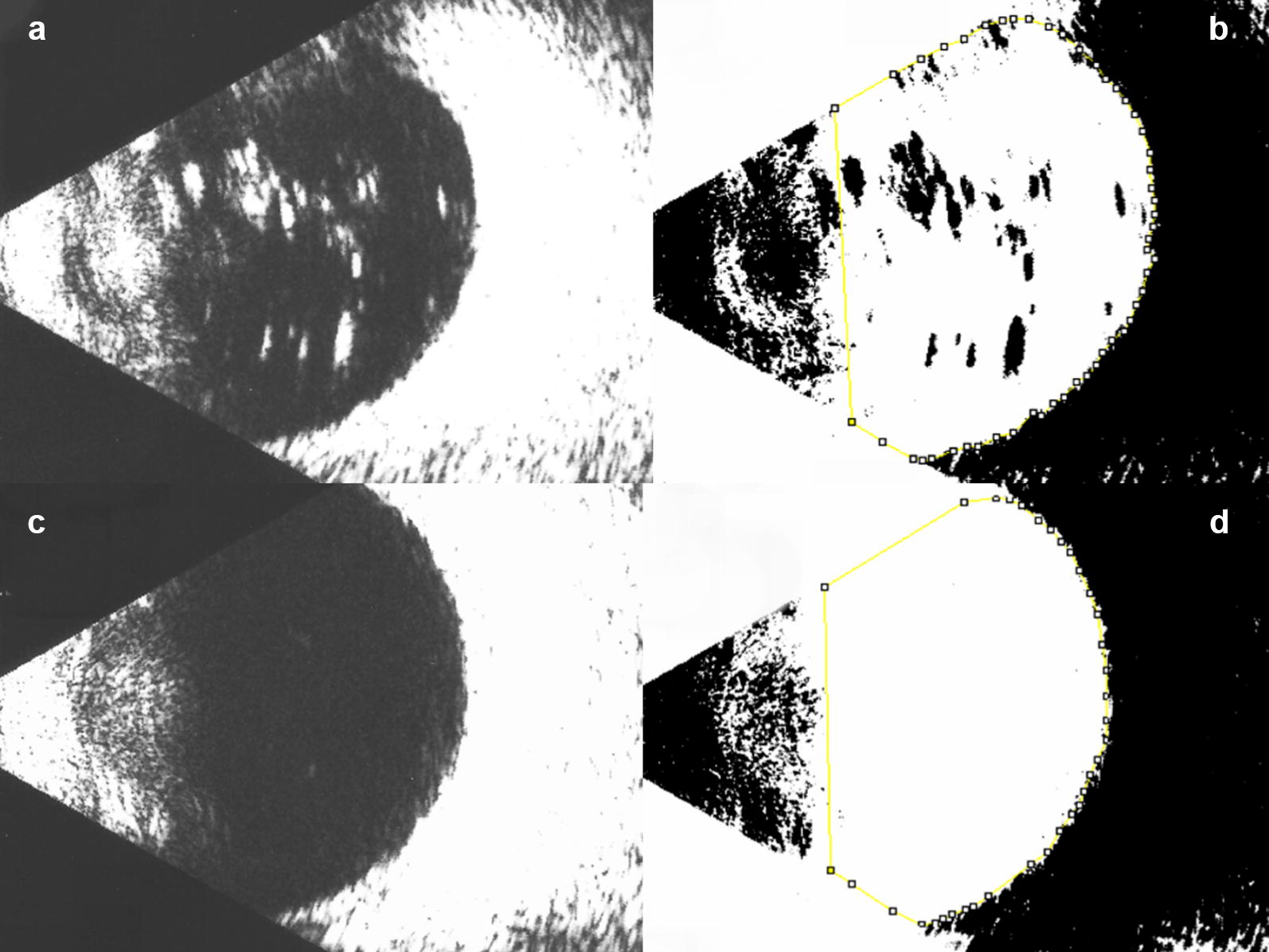
Illustration of the image processing using ImageJ to quantify silicone oil droplets. **a** (Top left): ultrasound image showing an eye previously treated the multiple injections presenting many hyperechoic areas. **b** (Top right): after binarization, a yellow line demarcates the vitreous borders. The presumed oil droplets are in black. **c** (Bottom left): ultrasound image of the contralateral, healthy eye of the same subject. **d** (Bottom right): after binarization of the control eye, only a few black dots can be seen within the demarcated vitreous area (yellow)

To quantify the residual silicone droplet objectively, a binarization method was applied to the B-mode echography images. The best image of each eye was displayed on a computer screen and evaluated by three masked graders independently (CSDJ, ALC, AGAF). Binarization of the B-mode echographic image was done by the default method. In summary, the B-mode image was analyzed by ImageJ (ImageJ version 1.52a; The National Institutes of Health, Bethesda, MD; available at: http://imagej.nih.gov/ij/). The area of the vitreous cavity was marked out from the image (Fig. 2). Then, it was binarized to emphasize the signals from the silicone oil using “default” in the “threshold”, a modified technique from previously reported [15]. ImageJ was used to determine the total area of the signals from the silicone oil and the vitreous. The index of silicone oil (IOS) was calculated as: area of signals from hyperechoic droplets/area of vitreous cavity × 100 (%).

In order to determine the factors significantly correlated with the amount of silicone oil, the correlations between IOS and the number of prior intravitreal injections were calculated.

### Statistical analysis

Data were analyzed with STATA 14.0 (StataCorp LP, College Station, TX, USA). Sample distribution was analyzed by the Shapiro–Wilk test. Agreement among the techniques to identify silicone oil droplets was assessed by Kappa’s coefficient. Multiple logistic regression was used for gender, age, prior treatment, number of injections and the lens status in association with the presence of silicone oil by both slit-lamp examination and ultrasonography. IOS obtained by 3 different graders was compared by Kruskal–Wallis test and the intra-class correlation (ICC) was calculated. Correlation between the number of previous injections and IOS was analyzed by Spearman’s correlation test. Finally, a direct comparison between treatment and control groups was performed using Wilcoxon test. For all analyses, a statistically significant level was set at 0.05.

## Results

Sixty-seven eyes (30 controls, 37 treated) of 34 subjects were included in this study. A descriptive analysis of the demographic data is presented in Table 1.

**Table 1:** Demographic data of the eyes included in this study

	Controls	Treated eyes	All
Gender			
Male	9 (30.00)	17 (45.95)	26 (38.81)
Female	21 (70.00)	20 (54.05)	41 (61.19)
Diagnosis			
Normal	17 (56.67)	0 (0.00)	17 (25.37)
AMD	8 (26.67)	14 (37.84)	22 (32.84)
DR	3 (10.00)	18 (48.65)	21 (31.34)
Venous occlusion	0 (0.00)	3 (8.11)	3 (4.48)
Other	2 (6.67)	2 (5.41)	4 (5.97)
Lens status			
Phakic	20 (66.67)	21 (56.76)	41 (61.19)
Pseudophakic	10 (33.33)	16 (43.24)	26 (38.81)
Age	70.53 ± 11.60	71.78 ± 10.85	71.22 ± 11.12
Mean number of injections	0.00 ± 0.00	9.30 ± 6.15	5.13 ± 6.50
Total	30 (100.00%)	37 (100.00%)	67 (100.00%)

*AMD* age-related macular degeneration, *BCVA* best-corrected visual acuity in logMAR, *DR* diabetic retinopathy

Slit-lamp examination found silicone oil droplets in 25 out of 37 (67.57%) treated eyes and in none of the control group. Similarly, ultrasonography identified silicone oil in 28 out of 37 (75.68%) treated eyes and in 1 out of 30 (3.33%) controls. The oil droplets were mostly found in the mid and upper areas of the anterior vitreous by slit-lamp examination. However, since the subjects were lying supine for ultrasonography, the droplets were found anteriorly, regardless of axial orientation of the B scan probe, suggesting that the oil droplets tend to float.

An observed agreement of 85.07% and a Cohen’s Kappa coefficient of 69.10% (p < 0.0001) between ultrasonography and biomicroscopy were found.

Considering only slit-lamp findings, multiple logistic regression disclosed an odds-ratio (OR) of 18.44 (95% confidence interval, 95% CI 0.60–560.03, p = 0.094) for silicone oil in previously treated eyes and 1.36 (95% CI 1.04–1.77, p = 0.024) for silicone oil according to the number of previous injections (Table 2). Considering ultrasound findings, multiple logistic regression disclosed an OR of 32.07 (95% CI 3.72–275.96, p = 0.002) for silicone oil in previously treated eyes and 1.11 (95% CI 0.95–1.29, p = 0.180) for silicone oil according to the number of previous injections (Table 2).

**Table 2:** Multiple logistic regression shows a statistically significant odds-ratio for the number of previous injections considering slit-lamp examination and for a prior treatment considering ultrasonography

	Slit-lamp			Ultrasonography		
	Odds ratio	95%-confidence interval	p	Odds Ratio	95%-confidence interval	p
Gender	0.617	0.111–3.423	0.581	0.327	0.076–1.415	0.135
Age	11.015	0.987–1.229	0.084	10.039	0.955–1.131	0.375
Prior treatment	184.436	0.607–560.030	0.094	320.706	3.727–275.967	0.002
Number of injections	13.607	1.042–1.777	0.024	11.096	0.953–1.292	0.180
Lens status	13.552	0.200–9.169	0.755	0.466	0.082–2.662	0.391
BCVA	0.414	0.063–2.719	0.359	0.240	0.050–1.152	0.075

*BCVA* best-corrected visual acuity in logMAR

**Fig. 3:**
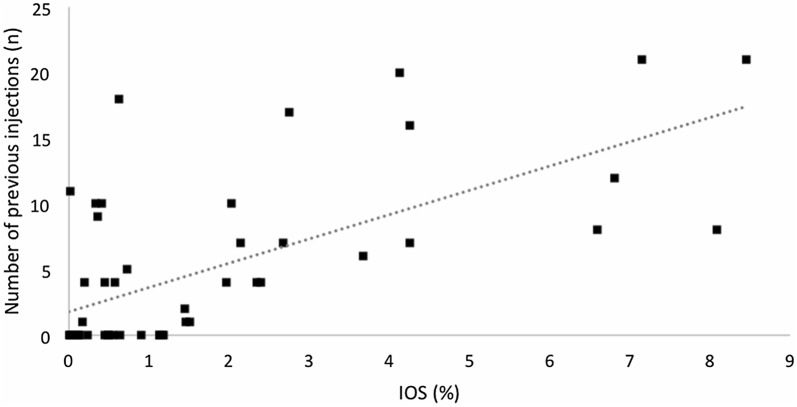
Graph showing that the greater the number of previous injections, the greater the index of silicone oil (IOS)

Kruskal–Wallis test showed no statistically significant difference of IOS among the 3 graders (p = 0.995). An intra-class correlation of 99.94% (95% CI 99.90–99.96%; p < 0.0001) was found for this variable. Wilcoxon test showed a statistically significant difference (p = 0.0006) between controls (0.41 ± 0.43%) and treated eyes (2.69 ± 2.55%). Spearman’s correlation test (0.61; p < 0.0001) showed that the greater the number of injections, the higher the IOS. Figure 3 shows the distribution of IOS according to the number of previous intravitreal injections.

## Discussion

We found silicone oil droplets in 68% to 76% of the eyes previously treated with intravitreal injections when assessed at the slit lamp or by ultrasonography, respectively. Identification of the silicone oil droplets was very straightforward at slit-lamp examination since they have a round and unique appearance. Tiny droplets might be mistaken for drug particles or vitreous debris. Whenever the examiners faced this situation and no clear droplet was seen, they classified the eye as negative.

Different studies have reported the presence of silicone oil droplets in the vitreous [6–8]. Bakri et al. found 15 eyes from a total of 1529 injections with presumed silicone oil droplets [6]. Khurana et al. have estimated the incidence of presumed silicone oil droplets in the vitreous cavity after intravitreal bevacizumab injection with insulin syringes to range from 0.03% (3230 injections) to 1.7% (3402 injections) at different time periods [8].

The aforementioned publications have an incidence of presumed silicone oil in the vitreous dramatically lower than the current study. Some reasons might help explain this difference. The syringes used in the United States are different from those used in Brazil. Therefore, some might release more oil than others. In fact, although it is not possible to state which syringe was used for each patient, the syringe models available in the last 5 years at the clinical setting where these patients were treated were Becton–Dickinson (BD) Plastipak 1 mL (Becton, Dickinson [BD] and Co., Curitiba, Brazil), SR 1 mL and BD SafetyGlide 1 mL (BD and Co., Holdrege, NE). Both the SR and the BD SafetyGlide syringes have been shown to release silicone oil droplets, especially after agitation [16]. Coincidently, the retina specialist responsible for those injections in this study used to flick the syringe until a few months prior to the data collection, which we believe promotes a greater detachment of oil from the inner surface of the syringes.

Flicking the syringe to dissociate fluid from air is common among retina specialists in their daily practice (personal communication). We first suspected that this was a problem when a cluster of six cases of inflammation following intravitreal injection of aflibercept developed [14]. All cases in this series had presumed silicone oil droplets in the vitreous. Additionally, all syringes had been agitated. A case–control analysis reinforced the suspicion that a new syringe introduced at the injection facility had an association with the cases with inflammation [14]. Thereafter, our group carried out experimental studies that showed that silicone oil might be released by syringes under steady-state conditions, but more commonly with agitation by flicking [16–18]. Additionally, needles have been shown to be coated with silicone oil [19].

Besides the suspected risk of inflammation, the presence of floaters secondary to silicone oil droplets in the vitreous can be so disturbing that vitrectomy might be required. The risks of complication of this procedure are not negligible, rarely resulting in blindness and/or legal action.

Quantification of residual silicone oil after vitreoretinal surgery by measuring the hyperechoic areas in comparison to the total area of the vitreous by ImageJ has been previously reported [15, 20]. In order to obtain a correlation between the number of prior injections and the presumed amount of oil, we used this software. The finding that a greater amount of oil was found with the increase in the number of previous procedures was as expected.

Although estimating the amount of silicone oil in the eye by ImageJ was reliably achieved, it also is a limitation of this study since a single biplanar scan was used to carry out this analysis. Therefore, the volume, per se, was not measured. Another limitation was the false-positivity by ultrasonography. Since one control eye (3%) was classified as positive, even without any history of intraocular procedures or pathologies, care should be taken when using this technique. It is possible that denser areas of the vitreous might have caused a false positive in one control subject. Further studies with a larger sample size are warranted in order to improve the ultrasonography technique for this purpose. Even so, the overall slit-lamp findings were quite similar and disclosed a good agreement with ultrasonography, which made the authors believe the results to be reliable and reproducible. Another limitation that should be taken into account is that the examiner responsible for the slit-lamp analysis was not masked to the study groups of each eye. However and interestingly, these findings were very similar to the ultrasonography ones, as aforementioned.

It should be remarked that the eyes receiving intravitreal antiangiogenic therapy were not categorized according the drug because 26 out of the 37 had been subjected to injection of 2 or more substances (data not shown). Although some retina specialists expect some drugs to be more associated with the release of silicone oil, our thoughts are that the syringes and the way they are handled are the key of this problem, and silicone oil droplets can be found regardless of the drug administered. Of note, aflibercept, bevacizumab, and ranibizumab are commercialized in vials (none preloaded in syringes) in Brazil. They are all aliquoted for equivalent syringes at the clinical setting of this study. It is another reason to consider there should be no difference in the prevalence of silicone oil droplets according to the drug.

In conclusion, silicone oil droplets were found in the majority of the eyes previously treated with antiangiogenic intravitreal injection. The greater the number of injections, the higher the likelihood of finding silicone oil; likewise, the greater the number of injections, the greater its amount can be found in the vitreous. An improvement in the technique of injection and better-quality syringes should be considered in order to minimize silicone oil droplets after intravitreal injection.

## Data Availability

The datasets used and/or analysed during the current study are available from the corresponding author on reasonable request
